# An Ancient Gene Network Is Co-opted for Teeth on Old and New Jaws

**DOI:** 10.1371/journal.pbio.1000031

**Published:** 2009-02-10

**Authors:** Gareth J Fraser, C. Darrin Hulsey, Ryan F Bloomquist, Kristine Uyesugi, Nancy R Manley, J. Todd Streelman

**Affiliations:** 1 Parker H. Petit Institute for Bioengineering and Biosciences and School of Biology, Georgia Institute of Technology, Atlanta, Georgia, United States of America; 2 Department of Ecology and Evolutionary Biology, University of Tennessee, Knoxville, Tennessee, United States of America; 3 Department of Genetics, University of Georgia, Athens, Georgia, United States of America; University of Helsinki, Finland

## Abstract

Vertebrate dentitions originated in the posterior pharynx of jawless fishes more than half a billion years ago. As gnathostomes (jawed vertebrates) evolved, teeth developed on oral jaws and helped to establish the dominance of this lineage on land and in the sea. The advent of oral jaws was facilitated, in part, by absence of hox gene expression in the first, most anterior, pharyngeal arch. Much later in evolutionary time, teleost fishes evolved a novel toothed jaw in the pharynx, the location of the first vertebrate teeth. To examine the evolutionary modularity of dentitions, we asked whether oral and pharyngeal teeth develop using common or independent gene regulatory pathways. First, we showed that tooth number is correlated on oral and pharyngeal jaws across species of cichlid fishes from Lake Malawi (East Africa), suggestive of common regulatory mechanisms for tooth initiation. Surprisingly, we found that cichlid pharyngeal dentitions develop in a region of dense hox gene expression. Thus, regulation of tooth number is conserved, despite distinct developmental environments of oral and pharyngeal jaws; pharyngeal jaws occupy hox-positive, endodermal sites, and oral jaws develop in hox-negative regions with ectodermal cell contributions. Next, we studied the expression of a dental gene network for tooth initiation, most genes of which are similarly deployed across the two disparate jaw sites. This collection of genes includes members of the ectodysplasin pathway, *eda* and *edar*, expressed identically during the patterning of oral and pharyngeal teeth. Taken together, these data suggest that pharyngeal teeth of jawless vertebrates utilized an ancient gene network before the origin of oral jaws, oral teeth, and ectodermal appendages. The first vertebrate dentition likely appeared in a hox-positive, endodermal environment and expressed a genetic program including ectodysplasin pathway genes. This ancient regulatory circuit was co-opted and modified for teeth in oral jaws of the first jawed vertebrate, and subsequently deployed as jaws enveloped teeth on novel pharyngeal jaws. Our data highlight an amazing modularity of jaws and teeth as they coevolved during the history of vertebrates. We exploit this diversity to infer a core dental gene network, common to the first tooth and all of its descendants.

## Author Summary

During evolution, teeth originated deep in the pharynx of ancient and extinct jawless fishes. Later, with the evolution of bony fish, teeth appeared in the mouth, as in most current vertebrates, although some living fishes retain teeth in the posterior pharynx. We integrate comparative morphology, paleontology, and molecular biology to infer the genetic control of the first dentition. We identify Hox genes as important components of an ancient dental gene-regulatory circuit and pinpoint subsequent modifications to this gene network that accompanied the evolution of toothed oral jaws. Furthermore, we highlight a set of genes conserved in the construction of all teeth, regardless of location and lineage. This core dental gene network is evolutionarily essential: nature appears never to have made a dentition without it.

## Abstract 3

A common gene regulatory circuit controls the development of all dentitions, from the original teeth in the throats of jawless fishes half a billion years ago, to the incisors and molars of modern vertebrates.

## Introduction

**Figure 1:**
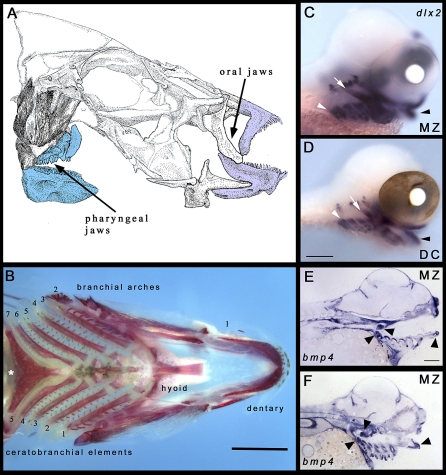
Malawi Cichlids Exhibit Toothed Oral and Pharyngeal Jaws (A) Schematic drawing (lateral view) of the generalized cichlid cranial skeleton, showing the relative location of the oral jaws (purple) and the pharyngeal jaws of PA7 (blue). (B) Dorsal view of an alizarin red skeletal preparation of the lower pharyngeal and oral elements of a juvenile D. compressiceps (DC) showing the series of branchial (pharyngeal) arches 1–7 and ceratobrachial elements CB1–5; the white asterisk indicates the toothed pharyngeal jaw. Scale bar represents 500 μm. (C and D) Lateral views with expression of *dlx2* labeling neural crest-derived cells in the pharyngeal arches of M. zebra [MZ] (C) and D. compressiceps [DC] (D). Both cichlids are 4 dpf and to the same scale; scale bar in (D) represents 200 μm. *dlx2* expression is observed throughout the arches from the mandibular arch, PA1 (black arrowheads), throughout the pharynx to the most posterior arch, PA7 (white arrowheads). *dlx2* expression is also present in neural crest-derived mesenchymal cells that populate the arches (white arrows). (E and F) Sagittal sections of MZ (5 dpf) showing expression of *bmp4* in multiple regions of the developing head and pharynx. *bmp4* is expressed throughout the arches in neural crest-derived arch mesenchyme for each pharyngeal arch (PA1–7), including both the mesenchyme and epithelial components of the developing teeth (black arrowheads). (E) The medial sagittal section and (F) lateral sagittal section show the gill-bearing arches (PA3–6). Both (E and F) are to the same scale; scale bar in (E) represents 200 μm.

Teeth are ancient vertebrate structures. During the early evolution of vertebrates, the appearance of a pharyngeal dentition greatly enhanced the capacity for processing food. Tooth-like structures located on elements of the pharyngeal series or skeleton were present in extinct jawless fishes (agnathans), for example members of the conodonts and later the thelodonts, which both possessed intricate, well-organized replacing dental systems [1–4]. Although tooth-like elements (denticles) were also present on the dermal surface of some agnathans (including thelodonts) and chondrichthyans, it was the occurrence of uniquely patterned pharyngeal teeth in agnathans that likely foreshadowed all other vertebrate oropharyngeal teeth [1,3–5]. Intriguingly, some extant fish still retain this ancient population of teeth in the posterior pharyngeal skeleton. More advanced groups of teleosts have adapted their posterior pharyngeal skeleton with teeth housed in discrete functional jaws, as in the cichlids and other groups [6–14] (Figure 1).

Teeth arise from a collaboration of different cell types that coalesce during the formation of the pharyngeal arches. Pharyngeal arches develop as a set of bulges on the ventrolateral side of the embryonic vertebrate head [15–17] (Figure 1). The formation of the pharyngeal arches involves the combination of all “germ” tissue layers: ectoderm covering each arch externally, endoderm lining the arches, and between these layers, the neural crest–derived mesenchyme surround a core of mesoderm [16]. Numerous common key developmental genes are required to regulate both arch patterning and development of the dentition (e.g., *bmp4* and *dlx2*; Figure 1C–1F). During the evolution of vertebrates, a general reduction in the number of pharyngeal arches is observed, from fossil agnathans (e.g., “ostracoderms” [18]) that possessed tens of arches and multiple (up to 45) gill openings [15,18], to amniotes that have five arches [15]. Teleost fish have seven pharyngeal arches [19]. The first pharyngeal arch (PA1) in the series forms the oral jaws (Figure 1). The second arch (PA2) forms the hyoid and the jaw support; the remaining posterior arches either contribute to the formation of the gills and gill-related skeletal structures (branchial) in fish or become incorporated into the throat of tetrapods [15]. The most posterior arch (PA7) in teleost fish houses a pharyngeal dentition, and in some groups, PA7 forms a second set of jaws, the pharyngeal jaws (Figure 1). The numbering of the arches PA1–7 in teleosts generally reflects the order of metameric development from anterior to posterior. However, the most terminal posterior arch (PA7 in teleost fish) develops out of series, ahead of most of the anterior arches [20].

The evolutionary origin of toothed oral jaws galvanized the dominance of gnathostomes and may have been prompted by the loss of Hox gene regulation in PA1 [21,22]. This notion has been supported by a report [21] of Hox gene expression during first arch formation in the lamprey (Lampetra fluviatilis), a jawless fish, although this observation is controversial (see Takio et al. [23]). All extant, jawed vertebrates do not express Hox genes in developing PA1. Numerous studies conclude that for correct first arch (PA1) fate, Hox genes must be absent, and consequently, for posterior arch fate, Hox genes must be present [24–28]. A branchial Hox code maintains the identity of more posterior pharyngeal arches, including the seventh pharyngeal arch (PA7) in teleosts [29] that house the terminal pharyngeal jaws.

Osteichthyan fish have retained the potential to form teeth throughout the oropharyngeal cavity, which includes the most posterior arch, PA7 (Figure 1). The ancestral condition for osteichthyans is teeth located throughout the oropharynx (e.g., Amia calva, the bowfin) [1,5]. However, an evolutionary trend toward a reduction in the sites that house teeth throughout the oropharynx is observed, similar to that of the arches themselves. For example, tetrapods have a dentition reduced to the oral jaws; further reductions are observed in tetrapods with complete (birds and turtles) or partial (mammals) loss of teeth within the single oral row. Even the developmental teleost model, the zebrafish (Danio rerio), has a reduced dentition with few teeth present on the lower PA7 and a complete loss of oral teeth [30,31].

Oral and pharyngeal teeth are assumed to be serially homologous [5,32,33]. This is thought to be the case despite the likelihood that tissue origins are not equivalent, with teleost oral teeth having the potential for ectodermal cell participation and pharyngeal teeth born out of endodermal epithelial tissue [1,5,34]. Tissue origin identification of the oral epithelium that contributes to tooth development has been consistently elusive. The break down of the stomodeum or the oropharyngeal/buccopharyngeal membrane leads to mixing of both anterior ectodermal and posterior endodermal cells within the oropharyngeal cavity, therefore a definite ectoderm/endoderm boundary may be unidentifiable. The mixed interface between the endoderm and ectoderm within the oropharyngeal cavity may be variable among vertebrate groups [35,36]. Reports of both histological and cell labeling evidence have suggested that some vertebrates develop oral teeth in close proximity to endodermal cells, even mammalian incisors [37] and molars (P. Sharpe, personal correspondence). Recently, Soukup et al. [36] observed that oral teeth of the Mexican axolotl form from epithelium either born of ectoderm, endoderm, or a mixture of the two, and teeth that form as a result of these specific cell types or their collaboration are indistinguishable. This therefore suggests that at least in the oral region, the origin of the epithelium may vary; the important combination for odontogenesis is some source of epithelium plus the underlying neural crest–derived ectomesenchyme [36]. The data of Soukup et al. [36] lead to the interpretation that most anterior oral teeth are likely ectodermal, posterior oral teeth develop from a mixed population of ectodermal and endodermal epithelia, and the most posterior teeth, such as those on PA7 in teleost fishes, are likely formed from strictly endodermal cells [1,5,34]. Isolated reports have concluded that the teeth on the oral and pharyngeal elements of teleost fish share expression of a small set of genes, with notable differences [31,38–41]. In addition, certain genetic factors, key to the developmental programming of the mammalian oral dentition, are similarly expressed in equivalent regions of the developing teleost pharyngeal dentition [31,38–44].

Despite the coordination of tooth and arch development (above), oral and pharyngeal odontogenesis is partly decoupled from associated bones and/or cartilage [20,22,30]. Mutations affecting the pharyngeal cartilages, including PA7, do not necessarily disrupt the development of pharyngeal teeth [20,30,45], and mutations affecting pharyngeal teeth do not necessarily disrupt cartilage development [46]. Interestingly, other zebrafish mutations that affect pharyngeal/branchial cartilage formation in most arches do not always affect the most posterior tooth bearing PA7 [20]. This suggests that PA7 has unique properties separating it from more anterior arches. The involvement of Hox genes during the development and organization of the pharyngeal skeleton [29] implies that pharyngeal teeth develop and fuse to skeletal elements in a Hox-positive environment, unlike those of the oral jaws that develop consistently in a Hox-negative region, unless the appropriate conditions for jaw formation regardless of location require a loss (albeit temporary, in the case of PA7) of Hox regulation.

The available data are thus equivocal on the molecular regulation of oral versus pharyngeal dentitions. These dentitions are evolutionarily decoupled; teeth arose first in the pharynx prior to the origin of jaws. They are functionally decoupled; many vertebrates possess pharyngeal teeth and not oral teeth (e.g., zebrafish), and many others possess oral teeth and not pharyngeal teeth (e.g., mammals). They are developmentally decoupled in space (PA1 vs. PA7), tissue distribution (contribution of ectoderm in PA1 vs. endoderm in PA7), and possibly by the influence of the Hox code. One of the major difficulties in interpreting available data is that they are drawn from organisms, often with only a single dentition (zebrafish or mouse), separated by vast evolutionary distances, or sampled species are taken from lineages exhibiting reduced dental diversity (e.g., medaka, trout) among close relatives.

**Figure 2:**
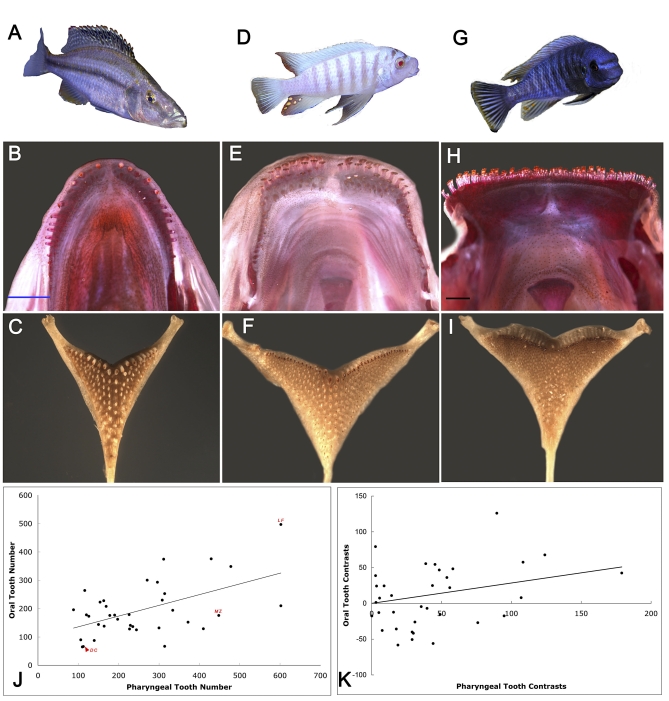
Oral and Pharyngeal Tooth Number Is Correlated in Malawi Cichlids (A–I) show three species of Malawi cichlid: D. compressiceps (DC) (A–C), M. zebra (MZ) (D–F), and L. fuelleborni (LF) (G–I). Dorsal views of (B, E, and H) show adult lower oral jaws, cleared and alizarin-stained bone/dentine preparation, and (C, F, and I) show adult lower (ceratobrachial [CB]5/PA7) pharyngeal jaws with the soft tissue removed. DC, MZ, and LF represent a range in oral and pharyngeal tooth number (Table S1): there are fewest teeth in DC, more teeth in MZ, many teeth in LF. (J) Across Malawi cichlids, a positive correlation is observed between the number of teeth on the oral and pharyngeal jaws (*r* = 0.53 without P. nigra and *r* = 0.66 including P. nigra; *p* < 0.00001), see (A–I). Data points labeled DC, MZ, and LF refer to the three species in (A–I). (K) Phylogenetically independent contrasts for tooth number across the Malawi cichlid flock without P. nigra (*r* = 0.39; *p* < 0.019; see Materials and Methods). Scale bars in (B) and (H) represent 500 μm. (E) and (H) are to the same scale. (C, F, and I) show the pharyngeal jaws to the same scale.

Our aim is to understand the relationships and constraints between evolutionarily, developmentally, and functionally decoupled oral and pharyngeal dentitions. Our models for this project are cichlid fishes from Lake Malawi, for three primary reasons. First, Malawi cichlids exhibit a tremendous diversity in oral and pharyngeal jaw dentitions, and this variation has evolved in a short evolutionary window [47]. Second, all cichlids possess modified posterior pharyngeal arches, which act as a functional jaw (Figure 1 and 2; [6,7]). Cichlids, and a few other teleost lineages [6–9,12–14,48], feature fusion of the bilateral units of the lower pharyngeal jaw cartilages (LPJ) and a novel muscular sling that pulls the LPJ upward to contact the hinged upper pharyngeal jaw units. This formidable pharyngeal machinery for food processing can produce enough force in some species to crush hard prey such as shelled molluscs [6,7]. Cichlid oral and pharyngeal jaws are evolutionarily and functionally decoupled [7,8]. Third, we have recently characterized a gene network (including *bmp2*, *bmp4*, *dlx2*, *eda*, *edar*, *pax9*, *pitx2*, *runx2*, *shh*, and *wnt7b*) associated with variation in oral jaw tooth row number, tooth number within rows, and the spacing of teeth [49]; we can therefore ask how this network of genes is expressed in dentitions on oral and pharyngeal jaws of the same organism. Here we use “gene network” in the sense of coordinated expression. Exact similarities between network topologies (e.g., interactions between nodes), while implicated, remain to be determined in each evolutionary lineage. We integrate these molecular data with comparative morphology and paleontology to (1) infer the ancient dental network used to pattern the first teeth and (2) suggest a core regulatory circuit common to all dentitions.

## Results

### Tooth Number Is Correlated across Jaws in Malawi Cichlids

We took advantage of oral and pharyngeal dental diversity among Lake Malawi cichlids to ask whether tooth number was controlled similarly on each jaw. We therefore estimated the number of teeth on both oral and pharyngeal jaws of adult fishes for a range of Malawi cichlid species spanning the major evolutionary lineages and the extremes of dental diversity (Figure 2). For instance, the large (0.5 m) pelagic predator Rhamphochromis esox possesses an average of 65 oral and 110 pharyngeal teeth, whereas the rock-dwelling algal brusher Petrotilapia nigra has on average 1,170 oral and 722 pharyngeal teeth (Figure 2; Table S1). There was a positive and highly significant correlation between the numbers of teeth on oral versus pharyngeal jaws (*r* = 0.53 without P. nigra and *r* = 0.66 including P. nigra; *p* < 0.00001; Figure 2J). This correlation is independent of evolutionary history as demonstrated by removing phylogenetic effects with independent contrasts (*r* = 0.39; *p* < 0.019; Figure 2K). These data indicate that regulators of tooth initiation (tooth number) are similar across the two dentitions, a surprise given functional independence, developmental differences, and evolutionary separation.

### Hox Genes Are Expressed in the Cichlid Pharyngeal Dentition

**Figure 3:**
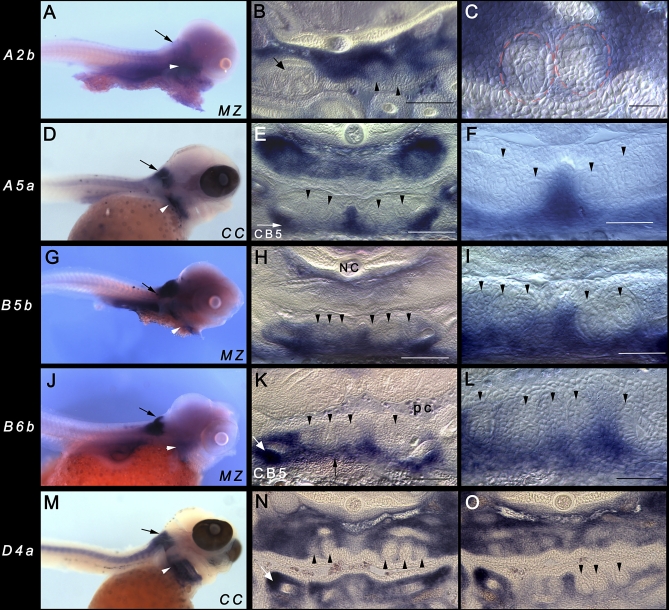
Multiple Hox Genes Are Expressed during Pharyngeal Jaw and Tooth Development in Malawi Cichlids (A–C) *hoxA2b* (*A2b*) expression in M. zebra (MZ). (D–F) *hoxA5a* (*A5a*) expression in C. conophorus (CC). (G–I) *hoxB5b* (*B5b*) expression in MZ. (J–L) *hoxB6b* (*B6b*) expression in MZ. (M–O) *hoxD4a* (*D4a*) expression in CC. (A, D, G, J, and M) show all whole-mount lateral views. (B, C, E, F, H, I, K, L, N, and O) show all coronal sections of the pharyngeal jaw (PA7/CB5). (A) *hoxA2b* is expressed in the hindbrain (black arrow) and the pharyngeal arch mesenchyme (white arrowhead). (B and C) show an oblique coronal section: (B) *hoxA2b* expression surrounds more mature upper pharyngeal dentition away from cells directly associated with the teeth (black arrow). Tooth germs at an earlier stage of development show the expression of *hoxA2b* in closer proximity within the mesenchymal cells enveloping the tooth germs (black arrowheads) and in (C) demarcated by red dashed circles. (D) *hoxA5a* is expressed in the hindbrain (black arrow) and in the posterior pharyngeal mesenchyme (white arrowhead). (E and F) show a coronal section: (E) *hoxA5a* expression underlying the teeth (black arrowheads) in the pharyngeal jaw (and in [F]). *hoxA5a* expression is present around the forming cartilages of the fifth ceratobranchial (CB5), (white arrow) and the upper fourth epi/pharyngobranchial. (G) *hoxB5b* is expressed in the hindbrain (black arrow) and the most posterior pharyngeal arch mesenchyme (white arrowhead). (H and I) show a coronal section: (H) *hoxB5b* marks the dental mesenchyme surrounding each tooth germ (black arrowheads) in the pharyngeal jaw (CB5); higher magnification is shown in (I). NC, notochord. (J) *hoxB6b* is present in the developing hindbrain (black arrow) and the posterior pharyngeal mesenchyme (white arrowhead). (K and L) show a coronal section: (K) *hoxB6b* expression surrounds each tooth in the dental mesenchymal cells (black arrowheads); stronger expression is observed at the base of each tooth unit (black arrow), possibly related to the attachment between the mineralized tooth and the underlying cartilage of CB5. Expression surrounds the pharyngeal cartilages (white arrow); a higher magnification is shown in (L). pc, pharyngeal cavity. (M) *hoxD4a* is expressed in the hindbrain (black arrow) and in the posterior pharyngeal mesenchyme (white arrowhead). (N and O) show a coronal section: (N) *hoxD4a* is strongly expressed in the dental mesenchyme surrounding each tooth germ of the pharyngeal jaw. Each individual tooth is demarcated by the mesenchymal expression (black arrowheads). *hoxD4a* is also strongly up-regulated in the mesenchyme directly enveloping the cartilages of CB5 (white arrow). Expression of *hoxD4a* is identical between the upper (N) and lower (O) pharyngeal dentition. (B, E, H, K, N, and O) are all to the same scale: scale bars represent 100 μm. Scale bar in (C) represents 20 μm. Scale bars in (F, I, and L) represent 50 μm. Embryos shown are 6–7 dpf.

Following (1) the correlation described above and (2) the idea that lack of Hox expression is permissive for toothed jaw development on PA1 [21], we predicted that Hox genes would be down-regulated during the development of the cichlid toothed pharyngeal jaw on PA7. We therefore examined the expression of seven Hox genes (*hoxA2b*, *hoxA5a*, *hoxB2a*, *hoxB5b*, *hoxB6b*, *hoxC6a*, and *hoxD4a*; Figure 3) in two cichlid species, Metriaclima zebra (MZ) and Copadichromis conophorus (CC) representing the two major Malawi evolutionary lineages [50], during a critical period when pharyngeal jaws and dentitions develop. Notably, all seven Hox genes were strongly expressed within the mesenchyme enveloping the pharyngeal jaw cartilages of PA7 with six of the seven genes examined (*hoxA2b*, *hoxB5b*, *hoxB6b*, and *hoxD4a*, Figure 3A–3C and 3G–3O; *hoxB2a* and *hoxC6a*; unpublished data) expressed in the dental mesenchymal cells directly surrounding the tooth germs. Furthermore, *hoxB5b* (Figure 3G–3I) and *hoxB6b* (Figure 3J–3L) are expressed in the basal dental mesenchyme within individual tooth germs (dental papilla) at this stage. *hoxA5a* (Figure 3D–3F) is the only Hox gene we examined not expressed in close proximity to the developing teeth, but is strongly expressed around the future regions of tooth attachment and cartilage maturation of both upper and lower elements of PA7 (Figure 3E and 3F). These data demonstrate, contrary to our prediction, that cichlid pharyngeal jaws and their dentitions develop in a Hox-positive environment.

### A Dental Regulatory Circuit Is Conserved across Oral and Pharyngeal Jaws

Tooth number is correlated on cichlid oral versus pharyngeal jaws (Figure 2J and 2K), but these jaws represent distinct cellular and developmental (Hox-negative vs. Hox-positive) environments (Figure 3). We therefore hypothesized that conservation in adult tooth pattern was due to conservation in a genetic network establishing tooth initiation on both jaws. We have recently described a dental gene network for cichlid oral jaws, a “periodic pattern generator” for interspecific variation in tooth row number, tooth number within rows, and tooth spacing [49]. Genes involved in this dental regulatory circuit include *bmp2*, *bmp4*, *dlx2*, *eda*, *edar*, *pax9*, *pitx2*, *runx2*, *shh*, and *wnt7b*; specific roles in odontogenesis have also been documented in the mouse (Mus musculus). A noted corollary of this hypothesis is that it might be surprising to observe the expression of ectodysplasin pathway genes *eda* and *edar* in the pharyngeal dentition (derived from endoderm), because these molecules are seemingly specified to ectodermal epithelial organs (see below), although expression has been observed in murine endoderm [51].

**Figure 4:**
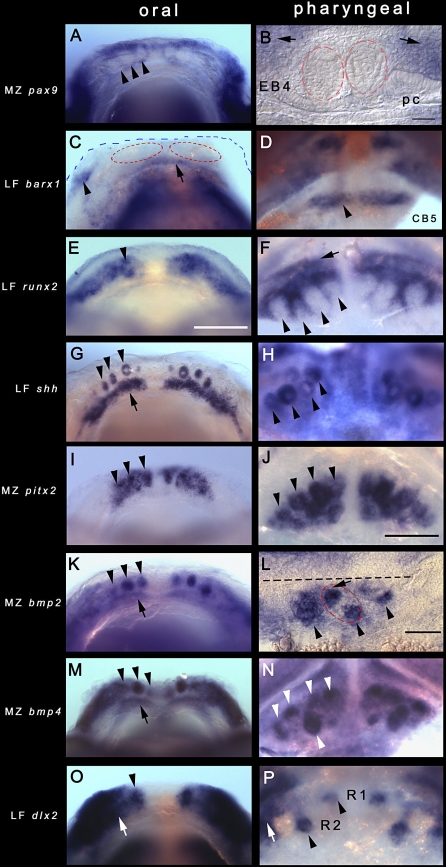
A Dental Gene Network Exhibits Conserved Expression in Oral and Pharyngeal Teeth (A, C, E, G, I, K, M, and O) show gene expression in the oral dentition, all dorsal views of the lower jaw. (D, F, H, J, N, and P) show dorsal views of the developing lower pharyngeal dentition; (B and L) are coronal sections through the pharyngeal teeth. M. zebra (MZ) (A, B, and I–N) and L. fuelleborni (LF) (C–H, O, and P) are represented here at the three to four oral tooth stage (∼6–7 dpf); the pharyngeal dentition develops ahead of the oral teeth, and so at this time point there are approximately seven pharyngeal teeth per pharyngeal quadrant. (A and B) show *pax9* expression in MZ. (A) In the lower oral jaw, *pax9* marks mesenchymal cells surrounding teeth (black arrowheads). (B) shows a coronal section showing upper pharyngeal teeth with *pax9* lateral to (black arrows) but not associated with teeth (red dashed circles). Scale bar in (B) represents 20 μm. EB4, epibranchial 4; pc, pharyngeal cavity. (C and D) show *barx1* expression in LF. (C) In the lower oral jaw (outlined in blue dashes), *barx1* is expressed in a band of mesenchymal cells (black arrow) lingual to the tooth sites (red dashed regions), and also in lateral cell clusters (black arrowhead) away from tooth sites. (D) *barx1* is localized to mesenchymal cells underlying tooth sites in developing lower pharyngeal jaw (CB5, black arrowhead). CB5, ceratobranchial 5. (E and F) show *runx2* expression in LF. (E) *runx2* labels both mesenchymal cells of the oral mesiodistal field for tooth competence and mesenchymal cells that surround the epithelial tooth germs (black arrowhead). (F) Equivalent expression is observed in the developing pharyngeal jaw (tooth germs, black arrowheads) and in tooth-competent regions of mesenchyme (black arrow). (G and H) show *shh* expression in LF. (G) *shh* is expressed in the epithelial cells of the developing oral dentition (black arrowheads; differences in expression from spots to open circles show variation in the stage of tooth development). *shh* also labels the epithelial odontogenic band for posterior tooth rows (black arrow). (H) *shh* is up-regulated in the pharyngeal dentition (black arrowheads) as in oral teeth. (I and J) show *pitx2* expression in MZ. (I) *pitx2* marks the dental-competent oral epithelium around the tooth sites and is up-regulated in tooth germs, from the thickened epithelium to the maturing tooth germ (black arrowheads). (J) Similarly, *pitx2* labels both dental competent epithelia and epithelial tooth germs (black arrowheads) of PA7/CB5. (K and L) show *bmp2* expression in MZ. (K) *bmp2* is localized to the epithelial tooth thickenings (black arrowheads) and the competent epithelia along the mesiodistal axis of the oral jaw. *bmp2* becomes coexpressed to both the developing epithelial (teleost enameloid cell cluster [TEC], black arrow in [L]) components and mesenchymal papilla of the tooth germs [49] and in epithelial cells in a band lingual to the first teeth (black arrow in [K]) for new tooth rows. (L) shows a coronal section of lower pharyngeal teeth; *bmp2* labels both the epithelial cells of the TEC (black arrow) and mesenchymal cells of the dental papilla (black arrowhead) of the same tooth (red dashed circle). In epithelial thickenings, *bmp2* is present in the thickened epithelial cells and is also expressed in the condensing cells of the underlying mesenchyme (black arrowheads). The black dashed line indicates the pharyngeal cavity. Scale bar in (L) represents 50 μm. (M and N) show *bmp4* expression in MZ. (M) In the oral jaw (OJ) and (N) the pharyngeal jaw (PJ), *bmp4* is expressed in dental epithelial cells of tooth germs (OJ, black arrowheads; PJ, white arrowheads); later, *bmp4* is coexpressed in the mesenchymal cells of the dental papilla. Along the mesiodistal axis of the oral jaw, *bmp4* labels the mesenchymal field of dental competence, and for new tooth rows lingually (black arrow). In addition, *bmp4* is expressed throughout the pharyngeal arch mesenchyme (see also Figure 1E and 1F). (O and P) show *dlx2* expression in LF. *dlx2* labels the first tooth (in the series) for each tooth row in both the oral jaw (PA1, black arrowhead in [O]) and the pharyngeal jaw (PA7, black arrowheads in [P]). *dlx2* is also present in the mesenchymal cells along the mesiodistal axis of the oral jaw and pharyngeal jaw (white arrows). (P) The black arrowheads show new row initiation. R1, row 1; R2, row 2. (A, C, E, G, I, K, M, and O) are all to the same scale. Scale bar in (E) represents 100 μm. (D, F, H, J, N, and P) are all to the same scale. Scale bar in (J) represents 100 μm. For details of expression in thin section for most of these markers, see [49].

Most of the genes analyzed (six of eight; *bmp2*, *bmp4*, *dlx2*, *pitx2*, *runx2*, and *shh*) have equivalent expression patterns in dental epithelium and/or mesenchyme during cichlid oral and pharyngeal tooth development (Figure 4). The two exceptions are provided by *pax9* and *barx1*. *pax9* is expressed within the developing dentition of the oral jaws (Figure 4A; [49]) but not in close proximity to developing teeth in the pharynx, although expression is noted in cells of the pharyngeal mesenchyme lateral to the teeth (similarly described in zebrafish [D. rerio], medaka [Oryzias latipes], and the Mexican tetra [Astyanax mexicanus] by Stock and colleagues [31]) but not associated with cells of the dental mesenchyme (Figure 4B). Conversely, *barx1* is not localized to the oral dentition. It is expressed in the flanks of the oral jaw outside of the tooth-forming region (Figure 4C). However, *barx1* is expressed in the pharyngeal mesenchyme underlying the dental epithelial thickenings of the pharyngeal teeth on CB5 (PA7) (Figure 4D).

### Ectodysplasin Pathway Genes Pattern the Endodermal Pharyngeal Dentition

**Figure 5:**
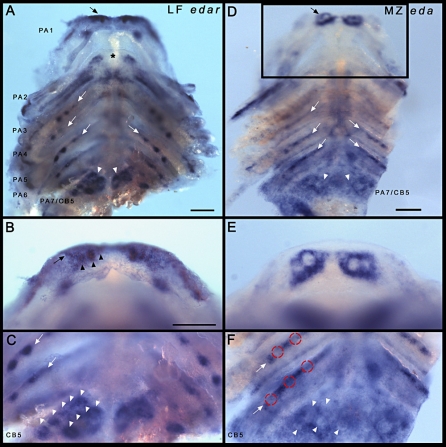
Ectodysplasin Pathway Genes, *eda* and *edar*, Are Expressed in Skeletal Structures throughout the Oropharynx **(**A, B, and C) show *edar* expression in the lower pharyngeal elements of L. fuelleborni (LF) (6 dpf); dorsal views. (A) shows *edar* expression in the entire lower pharyngeal series. *edar* is localized to the epithelium of the tooth germs (black arrow) of the oral jaws (B) (arrowheads), in the surrounding epithelium along the mesiodistal axis in (B) (arrow), and in the pharyngeal endoderm including the hyoid arch (asterisk in [A]). *edar* is expressed in gill raker bud epithelium (similar to tooth germs) on pharyngeal arches PA3–6 and in a band of gill raker initiation along the mesiodistal axes of each gill-bearing arch (white arrows in [A] and in [C]). *edar* also labels in the epithelial cells of the pharyngeal tooth germs on PA7 (white arrowheads in [A] and in [C]). (D, E, and F) show *eda* in the lower pharyngeal elements of M. zebra (MZ) (7dpf); dorsal views. (D) shows *eda* expression in the lower pharyngeal series of MZ. (D) is composed of two images of the same specimen; the boxed area of the oral jaw (separate image) is shown in (E). *eda* expression marks the mesenchymal cells surrounding and separating the tooth germs of the oral jaw (black arrow in [D]; open circles surrounded by *eda* expression represent the epithelial tooth germs; also see [49]), through to the equivalent cells surrounding the tooth germs of the pharyngeal jaw, PA7 (white arrowheads in [D] and in [F]). Between these separated dental sites, *eda* also labels the medial pharyngeal mesenchyme from the hyoid (PA2), including the second arch extension that will form the opercular flap (black arrowhead), through the series to the most posterior arch (PA7). *eda* is also expressed in relation to the initiating gill rakers lining each of the gill-bearing arches, PA3–6 (white arrows). Breaking up the bands of expression are *eda*-negative sites of gill raker bud initiation (red dashed circles) that express *edar* in (C). Scale bar in (B) represents 100 μm; (B, E, C, and F) are all to the same scale. Scale bars in (A and D) both represent 100 μm. CB5, ceratobranchial 5.

We observed the expression of ectodysplasin pathway genes, *eda* and its receptor *edar*, in conserved dental cell types on both oral and pharyngeal jaws. The ectodysplasin receptor, *edar*, is expressed in the epithelial thickenings and within the oral epithelial odontogenic band (OB), similar to *shh* and *pitx2* [49]. During morphogenesis, expression of *edar* remains confined to the epithelial tooth germ (Figure 5A–5C). *eda* is similarly expressed in both oral [49] and pharyngeal teeth, restricted to the mesenchymal cells directly surrounding the developing initial epithelial thickening (the mesenchymal “zone of inhibition” [ZOI]; [49]; Figure 5D–5F) and later during morphogenesis around the maturing tooth germs. This is in contrast to its expression in mammalian teeth where it is exclusively expressed in the epithelial cells of the intergerm space (ZOI) before its expression is recruited into the tooth during morphogenesis of the outer dental epithelium [52,53]. This is the first documentation of ectodysplasin pathway genes expressed in teeth likely derived from endoderm, deep within the pharyngeal/branchial arches. Our data complement a recent report that mutations in *eda* and *edar* result in loss of zebrafish pharyngeal teeth [46]. This result might have been expected due the expression of *Tabby-A* (*eda*) localized to the visceral and definitive endoderm in the mouse [51], although not related to an epithelial appendage per se (e.g., hair, tooth, or scale). *eda* and *edar* are members of the tumor necrosis factor (TNF) superfamily and are imperative for the correct formation and patterning of ectodermal appendages in vertebrates such as feathers, hair, teeth, scales, and glands [52–56]. Human mutations in these and other members of the ectodysplasin pathway cause various forms of hypohidrotic ectodermal dysplasia (HED), which manifests by specifically affecting ectodermal appendages [52].

### “Dental” Genes Similarly Pattern Skeletal Structures on PA3–6

**Figure 6:**
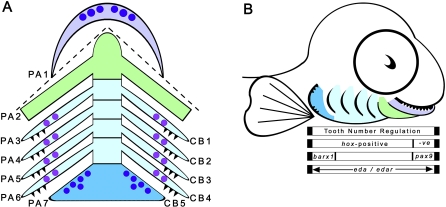
Teleost Pharyngeal Arches Exhibit Teeth on Old and New Jaws (A) Schematic diagram of the lower pharyngeal arches in an embryonic (∼7 dpf) cichlid fish (dorsal view). (B) Cartoon of the cichlid head showing pharyngeal arches (representing a general embryonic cichlid ∼7 dpf). (A and B) Oral jaw (PA1, lilac) houses a dentition (dark-blue circles) that develops in an ectoderm-influenced environment; dashed line in (A) represents the ectoderm/endoderm interface; a strict boundary may not exist due to cell mixing across this interface [36]. This dashed line also reflects the border between Hox gene–positive and Hox gene–negative oropharyngeal regions among vertebrates; we do not suggest a functional relationship between the two. The unique hyoid arch (PA2, green) is the only arch in the series that has no teeth (not a general rule for all fish) or gills/gill rakers; however, it does extend to cover all posterior arches as the opercular flap. PA3–6 (CB1–4, light blue) are gill-bearing arches (black triangles in [A]); these arches also house gill rakers (purple circles) that express a similar suite of genes during development in a similar temporospatial manner to the teeth on PA1 and PA7. Gill rakers will feature a secondary tooth/denticle set later in development. Oral and pharyngeal tooth development is generally governed by the same genetic regulators (see Figures 4 and 5); this corresponds to a positive correlation in the number of tooth units across the two disparate sites (tooth number regulation; see Figure 2). Pharyngeal tooth sites (PA7, blue) represent the first sites of tooth formation in vertebrates. The oral jaw and the teeth of PA1 develop devoid of hox gene expression (-ve), whereas the pharyngeal jaw and the teeth of PA7 (and all other arches in the series PA3–6) develop with hox genes strongly expressed in the pharyngeal mesenchyme around the forming pharyngeal jaw cartilages and teeth (hox positive; see Figure 3). *pax9* is only expressed in relation to the dentition of the oral jaw, whereas *barx1* is only expressed in relation to the dentition of the pharyngeal jaw (B). *eda* and *edar* are both expressed in a similar pattern throughout the entire pharyngeal arch series from the dentitions of the oral and pharyngeal jaw to the organization of the gill raker buds along the cartilage bars of PA3–6 (see Figure 5). Colors in (A) correspond to those in (B).

Both *eda* and *edar* are involved in “gill raker” patterning along the mesiodistal axis of each gill bar. Gill rakers are skeletal elements of the oropharyngeal cavity that line the dorsal region of the cartilaginous gill arches from PA3 to PA6 (Figure 5A, 5C, 5D, and 5F). Each gill/branchial arch (PA3–6) is defined by a band of *eda*/*edar* expression, from which *edar* is up-regulated at the site of initiation for gill raker primordia (Figure 5A–5C). *eda* is expressed during gill raker initiation similar to its expression in teeth (Figure 5E and 5F; [49]), labeling the interraker mesenchyme region for each gill raker primordium (Figure 5D and 5F). Thus, cichlid *eda* and *edar* are expressed in all seven arches, from the teeth and jaw of PA1 (oral jaw) throughout the series PA3–6 during gill raker placode formation, and in PA7, where they mark the pharyngeal dentition (Figures 5 and 6). Additionally, the non-dental, non–gill-bearing arch PA2 exhibits expression of the two ectodysplasin pathway genes in both internal pharyngeal endoderm and external arch ectoderm (Figure 5A and 5D), presumably recruited for the extension of PA2 to form the opercular flap gill cover in teleosts. The role of ectodysplasin pathway genes in the development of tetrapod PA2 remains unclear; in tetrapods PA2 skeletal elements support the jaw (hyoid) and contribute to the neck.

A collection of other dental markers (β*-catenin*, *bmp2*, *bmp4*, *dlx2*, *pitx2*, and *shh*; unpublished data) is also expressed in a similar manner during the patterning of the gill rakers. Gill rakers are iteratively initiated from a band of competence similar to the odontogenic band on the jaws, expressing these genes in a mesiodistal pattern, from which “raker buds” show localized expression. Furthermore, later in development, these elements house an additional set of teeth/denticles (unpublished data) [39,57,58]. Our data suggest that a conserved dental gene network periodically patterns distinct gill arch structures on PA3–6.

## Discussion

**Figure 7:**
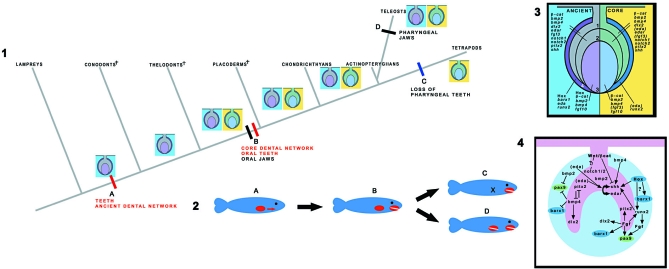
The Coevolutionary History of Jaws and Dentitions Panels 1 and 2 show a simplified evolutionary progression showing the hypothetical advent of the vertebrate ancient gene network (blue icon), core dental network (yellow icon), dentitions, and jaws. Point A indicates the origin of the ancient dental gene network and pharyngeal teeth in extinct (†) jawless fish. Point B indicates the origin of oral jaws in concert with the co-option of the core dental gene network allowing the development of teeth on the recently acquired oral jaws. This was accompanied by the loss of some genes occupying the ancient gene network, including the hox genes and *barx1* (*Barx1* was then regained in mammalian molar formation); Placoderms (the earliest jawed vertebrates) evolved an oral dentition independently in derived groups [74]; however, we suggest that the networks are common for teeth and were in place prior to the advent of oral jaws. Point C: the branch leading to the tetrapods shows a reduction in the sites that are occupied by a dentition and thus the pharyngeal dentition (ancient dental network) is lost (X in panel 2C). In addition to this loss, the oral dentition is greatly modified in tetrapods. Some vertebrates have lost the entire oral dentition (birds, turtles, etc.), and some have extreme modifications of the oral dentition (e.g., mammals). Point D: advanced groups of teleost fish, including the cichlids, have evolved a modified set of toothed pharyngeal jaws, further co-opting the ancient site of the first teeth and ancient dental gene network for involvement on a new functional jaw. Panel 2: schematic representation of the evolution of teeth and jaws in vertebrates. Panel 3: a schematic developing generalized tooth germ showing localization of the genes in the ancient vs. the core dental network (1, outer dental epithelium; 2, inner dental epithelium; 3, dental papillary mesenchyme; and 4, dental mesenchymal envelope). Color scheme is then represented on panel 1. Bracketed genes represent those with different cellular localization (mesenchyme or epithelium) in alternative vertebrates. Teleosts express *fgf3* in the dental epithelium, whereas mammalian *Fgf3* is mesenchymal; teleosts express *eda* in the mesenchyme, whereas mammalian *Eda* is epithelial. Panel 4: schematic representation of a generalized vertebrate tooth germ showing the putative interactions between the dental epithelial (pink) and dental mesenchymal (light blue) genetic players of the core dental network; those genes in blue ovals represent elements of the ancient dental network (e.g., Hox and *barx1*); those in green (*pax9*) represent molecules of neither the core nor the ancient dental network, present during oral dentitions of the mouse and cichlids (Table 1). *eda* is in brackets due to the differential expression between fish (mesenchyme) and mammals (epithelium). Regionalized signaling in an enamel organ such as that originating from the enamel knot has been purposely omitted from this generalized diagram. See Table S2 for references documenting each interaction.

There is avid interest in understanding the origin and developmental control of the dentition [1,3–5,39,59,60]. Teeth likely originated in the pharynx of jawless fishes that have long gone extinct (Figure 7). Ordered dentitions are an integral feature of gnathostome oral jaws; most extant gnathostome groups do not possess pharyngeal teeth. Some sharks and bony fishes do; pharyngognath teleosts possess toothed pharyngeal jaws [6–9]. Thus, cichlid fishes exhibit secondary jaws at the site of the first vertebrate dentition (in the pharynx) and teeth at the site of the first vertebrate jaws (PA1) (Figures 6 and 7). We find that tooth number is coordinately regulated on pharyngeal versus oral jaws across a broad diversity of Lake Malawi cichlids (Figure 2). Despite differences in the ecological function of these toothed jaws and despite differences in their developmental environment (Hox-negative, ectodermal contribution vs. Hox-positive, endodermal), conserved patterns of tooth initiation appear to be controlled by common gene regulatory circuitry. We interpret these data to hold important implications for the first vertebrate dentition, and the origin of an ancient dental gene network, retained in pharyngeal endoderm of modern fishes and modified for teeth on oral jaws (Figures 6 and 7).

**Table 1:**
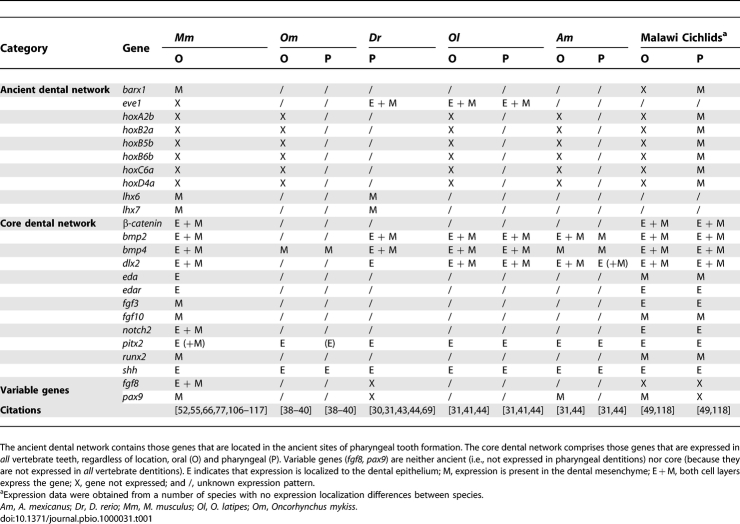
Identifying the Core versus Ancient Dental Network across Vertebrates

### Was Hox Expression Present in the Ancestral Dentition?

Our data demonstrate that Hox genes are expressed in cichlid pharyngeal jaws as the pharyngeal dentition initiates. Moreover, expression of a subset of these genes is observed within dental mesenchyme (*hoxA2b*, *hoxB5b*, *hoxB6b*, and *hoxD4a*, Figure 3; and *hoxB2a* and *hoxC6a*; unpublished data). Hox expression in teeth has not been noted before. This is not surprising; the majority of vertebrate developmental models do not possess a pharyngeal dentition (Hox genes are not expressed in PA1), and zebrafish Hox expression [27,61] has not been assessed during pharyngeal odontogenesis. This observation prompts a series of related questions: what role, if any, do Hox genes play in the pharyngeal dentition? Did ancient pharyngeal teeth express Hox genes? Hox genes (or their absence) are neither necessary nor sufficient for tooth initiation. All vertebrate oral dentitions develop in a Hox-negative environment. Initiation of the mouse dentition is unaffected when *Hoxa2* is overexpressed in the first arch [22]. Additionally, tooth development proceeds normally when Hox-positive mesenchyme from the second arch is recombined with first arch epithelium [22]. These latter results may have more to say about the independence of teeth and the jaws that house them (also [24–26]) than about the role of Hox genes in a developing tooth.

Notably, in other organ (e.g., limb) regulatory networks [62], Hox genes are upstream of a number of dental markers (*barx1*, *bmp2*, *bmp4*, *dlx2*, *pitx2*, and *shh*), and as such, Hox regulation might affect later aspects of pharyngeal tooth morphogenesis, replacement, or shape. One putative Hox target, *barx1* [62], is expressed in cichlid pharyngeal, but not oral, dentitions (Figures 4 and 6). *Barx1* is downstream of Fgf8 in mouse molars [63,64]; *fgf8* is absent from the oral and pharyngeal dentitions of all fishes examined to date [43,49]. Therefore, it is intriguing to speculate that Hox and *barx1* expression were components of an ancient dental program deep in the pharynx of jawless fishes, retained in the pharyngeal teeth of extant fishes. Hox genes may have played a patterning role for these first teeth, which lined an endoderm-rich pharyngeal cavity devoid of bony jaw elements. We speculate that as gnathostomes evolved a Hox-negative oral jaw, *barx1* expression was initially absent in oral teeth, and later recaptured by Fgf8 in mammalian molars. *pax9*, a paired domain (not homeodomain) transcription factor, may have replaced Hox expression in gnathostome oral dental mesenchyme. *pax9* is not essential for vertebrate tooth development (it is not expressed in all teeth), although it is necessary for mammalian odontogenesis [65,66].

### The Origin of an Ancient Dental Network and Deployment on Old and New Jaws

We propose that an ancient dental gene network constructed the first tooth-like structures deep within the pharyngeal arches of jawless fishes, more than half a billion years ago [1–4,59,67,68]. This ancient dental regulatory circuit has been conserved in modern fishes as those markers expressed in pharyngeal dentitions. This dental network is comprised of genes present during pharyngeal tooth initiation: *barx1*, *bmp2*, *bmp4*, *dlx2*, *pitx2*, *runx2*, and *shh*, including the ectodysplasin pathway genes, *eda* and *edar*, with a contribution from Hox molecules. In addition to genes described here, a number of others expressed in the pharyngeal dentition of teleosts are part of the ancient dental network, including *eve1* [41,69], *lhx6*, and *lhx7* [43] (Figure 7; Table 1). β*-catenin*, *fgf3*, *fgf10*, and *notch2*, a set of stem cell markers recruited during cichlid oral jaw tooth replacement (G. J. Fraser and J. T. Streelman, unpublished data) are also assigned to the ancient dental network, based on expression in pharyngeal teeth (Figure 7; Table 1).

We hypothesize that this ancient dental network has patterned all pharyngeal teeth, from the first dentitions in now-extinct jawless vertebrates to modern osteichthyan and chondrichthyan fishes. Although an ambiguous relationship exists between the homology of the elements of the dermal skeleton and teeth/denticles of the oropharynx [68], we envisage a plausible scenario that follows the general “inside-out” model of odontode evolution [1,3,4,59] in which pharyngeal endoderm in collaboration with neural crest–derived ectomesenchyme permitted the development of the first discrete, patterned dental units in jawless vertebrates. In contrast, the “outside-in” notion of vertebrate odontode evolution [1,3,4,59,70], that dermal denticle units like those of modern elasmobranchs (sharks and rays) “migrated” into the mouth cavity coinciding with the appearance of oral jaws, is confidently contested as the earliest “toothed” vertebrates (i.e., conodonts) lacked a dermal skeleton. Thus, it seems that pharyngeal teeth were the progenitor population for all vertebrate dentitions. We therefore propose that this ancient dental network was established close to the origin of vertebrates and was adopted for the formation of the first teeth.

This regulatory network was later co-opted and modified (Figure 7) to form teeth on the first jaws of gnathostomes (the oral jaws, PA1), providing the prerequisite for extreme predatory feeding, in the absence of Hox gene expression [21,71]. We infer from these data that the transition from agnathans to the first gnathostomes coincided with further modifications of the dental network governing the development of the early oral dentition. We observe a number of genes with variable dental expression patterns between vertebrates. *pax9* (Pax9) is imperative for mammalian tooth development, expressed during cichlid (Figures 4 and 6 and Table 1; [49]) and Mexican tetra [31] oral tooth initiation, but not expressed in relation to the pharyngeal teeth of cichlids (Figure 4) and zebrafish [43]. This suggests that *pax9* was not part of the ancient dental network but became a player in Hox-negative oral tooth evolution. *Fgf8* is an important regulator of murine odontogenesis, retained during the establishment of a potential avian odontogenic cascade [72], but is not present during the development of any teleost dentition [43,49]. Thus, we can assume Fgf8 was recruited in collaboration with tooth development during tetrapod dental evolution. In addition to these modifications, a member of the ancient dental network (present in pharyngeal teeth), *barx1*, not involved in oral tooth development in fish (Figure 4) was subsequently adopted for mammalian molar formation. Some genetic participants of tooth development that made the transition from the ancient gene network to the evolution of oral teeth, e.g., *eve1* [41,69], are not involved in tetrapod odontogenesis. Taken together, these reports of variable dental genes (*pax9*, *fgf8*) and members of the ancient gene network that have been lost (and regained) in oral dentitions (*barx1*, *eve1*) across vertebrates suggest that they are not “*evolutionarily essential*” for tooth development. The first oral dentitions during the advent of gnathostomes likely developed in a transitional genetic and cellular environment as the consequences of major changes in cell signaling (e.g., loss of Hox) sorted. Thus it is intriguing to note lack or the simplified peripheral oral dentition in the first gnathostomes (e.g., derived placoderms [73–75]), an experimental dental transition.

Subsequently in some groups of advanced teleost fishes [6–9], including cichlids, the ancient dental network located on PA7, in coordination with a recent adaptation of the pharyngeal skeleton, led to the evolution of a new functional toothed jaw, the pharyngeal jaw. Thus, the ancient dental gene network, once used for the first teeth in the pharynx of extinct jawless vertebrates, has been deployed on an entirely novel set of jaws (Figures 1, 2, and 7).

### The Core Dental Regulatory Network

Our analysis identifies a number of genes expressed commonly on cichlid oral and pharyngeal jaws (Figures 4 and 5). Many of these are similarly employed in the pharyngeal dentition of zebrafish, across oral and pharyngeal dentitions in the rainbow trout, Japanese medaka, and Mexican tetra, and in the oral jaws of tetrapods (Table 1 and references therein). These common patterns define a core dental regulatory network likely expressed in the first tooth and all of its evolutionary descendants, regardless of anatomical location within the oropharynx. By definition, core genes are part of the ancient dental network, but not necessarily vice versa.

*shh* is a core marker of dental epithelial initiation, as is *pitx2*, *bmp2*, *edar*, and to some degree, *bmp4*, *dlx2*, and *eda*. In response to initial epithelial signals [76], molecules within the neural crest-derived ectomesenchyme activate the collaboration between these cell layers toward morphogenesis of the unit tooth; mesenchymal instigators of tooth development include *bmp2*, *bmp4*, *dlx2*, *runx2*, and *eda* (with *eda* deployment variable between vertebrates [49] although its role is potentially equivalent; Table 1). β*-catenin*, *fgf3*, *fgf10*, and *notch2* are active during the initiation of dentitions and are recruited similarly in the dental stem cell niche of cichlid replacement teeth (G. J. Fraser and J. T. Streelman. unpublished data) and in continuously growing mouse incisors [77–79]. The core dental network represents a conserved set of molecules for tooth development that provides the molecular machinery and developmental constraints for all teeth, regardless of cellular origin (endodermal or ectodermal) or Hox gene contribution. We suggest that this core set is *evolutionarily essential*; no known examples of correctly patterned dentitions occur without the involvement of core genes. It is likely that nature has never made a tooth without this core genetic network. It is notable that members of the core dental network are coexpressed in the development of other vertebrate organs such as scales, feathers, and hairs [54,56,80–86] and that the origin of these gene families predate vertebrates altogether. Regulatory interactions among the core genes are themselves likely to be ancient, and therefore evolutionarily successful. Such ancient developmental regulatory networks may be particularly robust to failure (for instance, null mutations in human, dog, and cow ectodysplasin pathway genes affect morphogenesis but usually do not lead to loss of all teeth [87–89]) while retaining the capacity for evolvability [46,49,52,90,91].

It is impossible to study the developmental programs that controlled morphologies of extinct organisms. It is possible, however, to infer evolutionary transitions from modern phenotypic diversity through to origins [92–98]. Here, we have combined paleontology, molecular developmental biology, and comparative morphology to infer the developmental basis of ancient dental structures close to the origin of vertebrates and their evolutionary progression through time to recent diversity.

## Materials and Methods

### Phylogenetic analysis.

To generate a phylogenetic tree for the species examined, we assembled published ND2 data from a total of 37 species of Lake Malawi cichlids and several outgroup species. Modeltest 3.06 [99] was used to identify the best model of molecular evolution for each codon site. With the ND2 gene partitioned into its codon sites, Bayesian analyses were executed to find approximations of the maximum likelihood tree using MrBayes 3.0 [100] with methods similar to those described in Hulsey et al. [101].

### Comparative analyses of correlation in tooth number.

Pharyngeal tooth counts were performed on high-magnification images of Malawi cichlid lower pharyngeal jaws, and each tooth was counted (see Figure 2). We counted all lower oral teeth for a number of specimens and devised a system to estimate oral tooth number that replicated the counts. Oral tooth number was estimated based on the exact numbers of teeth on one half of the lower jaw first row, multiplied by the exact number of rows, multiplied by 2 (for the two halves of the oral jaw process; Figure 2). To assess the putative relationship among oral jaw and pharyngeal lower jaw tooth number, we first examined the correlation between Malawi species values. Between one and four individuals (70 specimens total) were documented per 37 species (Table S1); the correlation was then based on the mean values. However, because species are not evolutionarily independent [102], we also performed an independent contrast analysis. For the phylogenetic backbone of this analysis, we used the single best ND2 phylogeny recovered above. Many of the species relationships were recovered only as polytomies due to a lack of base pair changes among these recently diverged species [103]. Phylogenetic independent contrast analyses were then performed in order to assess the independent contrast correlations among the number of teeth on each jaw. The “crunch” algorithm was used in CAIC because it treats all variables as continuous. Analyses were run with untransformed tooth number because the correlation of species values suggested the variance was equal for species possessing both large and small numbers of teeth. Because tooth number may increase over ontogeny (but see [104]), we also examined the correlation of tooth number on the two jaws with log_10_ specimen standard length (SL) used as a phylogenetic covariate. P. nigra is a species with extremely large numbers of teeth on both the oral and pharyngeal jaws; thus the inclusion of P. nigra may have disproportionate affects on the regression trend. We therefore examined, and report the species level correlation with and without P. nigra.

### Fish husbandry.

Embryos and fry of multiple species of Lake Malawi cichlids (Copadichromis conophorus [CC], Dimidiochromis compressiceps [DC], Metriaclima zebra [MZ], and Labeotropheus fuelleborni [LF]) were raised to the required stage in a recirculating aquarium system (GIT) at 28 °C. Embryo ages (in days postfertilization [dpf]) were set after the identification of mouth brooding females (day 0). Embryos were then removed from the mouths of brooding females and, if required, were maintained for further development in separate culture tanks at 28 °C. All animals were handled in strict accordance with good animal practice as defined by the relevant national and/or local animal welfare bodies, and all animal work was approved by the appropriate committee at Georgia Institute of Technology.

### Sequences.

Cloned sequences used to generate digoxigenin-labeled antisense riboprobes from Malawi cichlid species have been published [49], additional sequences have been deposited in GenBank (http://www.ncbi.nih.nlm.gov; accession numbers FJ594754–FJ594761 and FJ597647). Many genes were identified through partial genome assemblies of L. fuelleborni and M. zebra [50] and cloned from M. zebra and L. fuelleborni cDNA libraries, including all of the Hox genes present in this study (cichlid Hox sequences from genomic contigs are also published in [105]). Overall, Malawi cichlids exhibit almost no sequence divergence; the average nucleotide diversity for comparisons across the Malawi assemblage is 0.26%, less than among laboratory strains of the zebrafish [50].

### In situ hybridization.

In situ hybridization experiments were based on a protocol from [49] and references therein. Specimens for in situ hybridization were anesthetized in tricaine methanesulfonate (MS222; Argent) and fixed overnight in 4% paraformaldehyde (PFA) in 0.1% phosphate-buffered saline (PBS) at 4 °C. Specimens were stage-matched based on external features, including pectoral and caudal fin development and eye development and maturity. All in situ hybridization experiments were performed with multiple specimens (multiple individuals were fixed at regular intervals, within single broods, then experiments were repeated at least twice with alternative broods) to fully characterize the expression patterns. After color reaction (NBT/BCIP; Roche) embryos were washed in PBS and fixed again in 4% PFA, before whole-mount imaging using a Leica Microsystems stereo microscope (MZ16). Embryos for sectioning were embedded in gelatin and chick albumin with 2.5% gluteraldehyde. The gelatin-albumin blocks were postfixed in 4% PFA before sectioning. Thin sections were cut at 15–25 μm using a Leica Microsystems VT1000 vibratome.

## Supporting Information

Table S1 Mean Oral and Pharyngeal Tooth Counts and Standard Length (SL) for One to Four Malawi Cichlid Adults from 37 Species D. compressiceps, M. zebra, and L. fuelleborni (asterisks) are indicated on Figure 2A–2K.
(30 KB DOC)

Table S2 Referenced Molecular Interactions during Tooth Development from the Interactions Schematized in Figure 7, Panel 4. (80 KB DOC)

## Abbreviations

PA: pharyngeal arch

## References

[pbio-1000031-b001] Smith MM (2003). Vertebrate dentitions at the origin of jaws: when and how pattern evolved. Evol Dev.

[pbio-1000031-b002] Donoghue PC (2001). Microstructural variation in conodont enamel is a functional adaptation. Proc Biol Sci.

[pbio-1000031-b003] Smith MM, Coates MI, Teaford MF, Smith MM, Ferguson MWJ (2000). Evolutionary origins of teeth and jaws: developmental models and phylogenetic patterns. Development, function and evolution of teeth.

[pbio-1000031-b004] Smith MM, Coates MI, Ahlberg PE (2001). The evolution of vertebrate dentitions: phylogenetic pattern and developmental models (palaeontology, phylogeny, genetics and development). Major events in early vertebrate evolution.

[pbio-1000031-b005] Stock DW (2001). The genetic basis of modularity in the development and evolution of the vertebrate dentition. Philos Trans R Soc Lond B Biol Sci.

[pbio-1000031-b006] Hulsey CD (2006). Function of a key morphological innovation: fusion of the cichlid pharyngeal jaw. Proc Biol Sci.

[pbio-1000031-b007] Hulsey CD, Garcia de Leon FJ, Rodiles-Hernandez R (2006). Micro- and macroevolutionary decoupling of cichlid jaws: a test of Liem's key innovation hypothesis. Evolution.

[pbio-1000031-b008] Liem KF (1973). Evolutionary strategies and morphological innovations: cichlid pharyngeal jaws. Syst Zool.

[pbio-1000031-b009] Tibbetts IR, Carseldine L (2003). Anatomy of a hemiramphid pharyngeal mill with reference to Arrhamphus sclerolepis krefftii (Steindachner) (Teleostei: Hemiramphidae). J Morphol.

[pbio-1000031-b010] Mehta RS, Wainwright PC (2007). Raptorial jaws in the throat help moray eels swallow large prey. Nature.

[pbio-1000031-b011] Mehta RS, Wainwright PC (2007). Biting releases constraints on moray eel feeding kinematics. J Exp Biol.

[pbio-1000031-b012] Carr A, Tibbetts IR, Kemp A, Truss R, Drennan J (2006). Inferring parrotfish (Teleostei: Scaridae) pharyngeal mill function from dental morphology, wear, and microstructure. J Morphol.

[pbio-1000031-b013] Streelman JT, Karl SA (1997). Reconstructing labroid evolution with single-copy nuclear DNA. Proc Biol Sci.

[pbio-1000031-b014] Mabuchi K, Miya M, Azuma Y, Nishida M (2007). Independent evolution of the specialized pharyngeal jaw apparatus in cichlid and labrid fishes. BMC Evol Biol.

[pbio-1000031-b015] Graham A (2008). Deconstructing the pharyngeal metamere. J Exp Zoolog B Mol Dev Evol.

[pbio-1000031-b016] Graham A (2001). The development and evolution of the pharyngeal arches. J Anat.

[pbio-1000031-b017] Graham A (2003). Development of the pharyngeal arches. Am J Med Genet A.

[pbio-1000031-b018] Janvier P, Desbiens S, Willett JA, Arsenault M (2006). Lamprey-like gills in a gnathostome-related Devonian jawless vertebrate. Nature.

[pbio-1000031-b019] Hulsey CD, Fraser GJ, Streelman JT (2005). Evolution and development of complex biomechanical systems: 300 million years of fish jaws. Zebrafish.

[pbio-1000031-b020] Schilling TF, Piotrowski T, Grandel H, Brand M, Heisenberg CP (1996). Jaw and branchial arch mutants in zebrafish I: branchial arches. Development.

[pbio-1000031-b021] Cohn MJ (2002). Evolutionary biology: lamprey Hox genes and the origin of jaws. Nature.

[pbio-1000031-b022] James CT, Ohazama A, Tucker AS, Sharpe PT (2002). Tooth development is independent of a Hox patterning programme. Dev Dyn.

[pbio-1000031-b023] Takio Y, Pasqualetti M, Kuraku S, Hirano S, Rijli FM (2004). Evolutionary biology: lamprey Hox genes and the evolution of jaws [comment]. Nature.

[pbio-1000031-b024] Alexandre D, Clarke JD, Oxtoby E, Yan YL, Jowett T (1996). Ectopic expression of Hoxa-1 in the zebrafish alters the fate of the mandibular arch neural crest and phenocopies a retinoic acid-induced phenotype. Development.

[pbio-1000031-b025] Pasqualetti M, Ori M, Nardi I, Rijli FM (2000). Ectopic Hoxa2 induction after neural crest migration results in homeosis of jaw elements in Xenopus. Development.

[pbio-1000031-b026] Grammatopoulos GA, Bell E, Toole L, Lumsden A, Tucker AS (2000). Homeotic transformation of branchial arch identity after Hoxa2 overexpression. Development.

[pbio-1000031-b027] Hunter MP, Prince VE (2002). Zebrafish hox paralogue group 2 genes function redundantly as selector genes to pattern the second pharyngeal arch. Dev Biol.

[pbio-1000031-b028] Baltzinger M, Ori M, Pasqualetti M, Nardi I, Rijli FM (2005). Hoxa2 knockdown in Xenopus results in hyoid to mandibular homeosis. Dev Dyn.

[pbio-1000031-b029] Miller CT, Maves L, Kimmel CB (2004). moz regulates Hox expression and pharyngeal segmental identity in zebrafish. Development.

[pbio-1000031-b030] Yelick PC, Schilling TF (2002). Molecular dissection of craniofacial development using zebrafish. Crit Rev Oral Biol Med.

[pbio-1000031-b031] Stock DW, Jackman WR, Trapani J (2006). Developmental genetic mechanisms of evolutionary tooth loss in cypriniform fishes. Development.

[pbio-1000031-b032] Stock DW, Weiss KM, Zhao Z (1997). Patterning of the mammalian dentition in development and evolution. Bioessays.

[pbio-1000031-b033] Donoghue PJC (2002). Evolution of development of the vertebrate dermal and oral skeletons: unraveling concepts, regulatory theories, and homologies. Paleobiology.

[pbio-1000031-b034] Edwards LF (1929). The origin of the pharyngeal teeth of the carp (Cyprinus carpio Linnaeus). Ohio J Sci.

[pbio-1000031-b035] Soukup V, Epperlein H-H, Horacek I, Cerny R (2007). Oral morhogenesis in axolotl and the first evidence of oral endodermal teeth for gnathostomes. Eur Cell Mater.

[pbio-1000031-b036] Soukup V, Epperlein HH, Horacek I, Cerny R (2008). Dual epithelial origin of vertebrate oral teeth. Nature.

[pbio-1000031-b037] Imai H, Osumi N, Eto K (1998). Contribution of foregut endoderm to tooth initiation of mandibular incisor in rat embryos. Eur J Oral Sci.

[pbio-1000031-b038] Fraser GJ, Berkovitz BK, Graham A, Smith MM (2006). Gene deployment for tooth replacement in the rainbow trout (Oncorhynchus mykiss): a developmental model for evolution of the osteichthyan dentition. Evol Dev.

[pbio-1000031-b039] Fraser GJ, Graham A, Smith MM (2006). Developmental and evolutionary origins of the vertebrate dentition: molecular controls for spatio-temporal organisation of tooth sites in osteichthyans. J Exp Zool B: Mol Dev Evol.

[pbio-1000031-b040] Fraser GJ, Graham A, Smith MM (2004). Conserved deployment of genes during odontogenesis across osteichthyans. Proc R Soc Lond B Biol Sci.

[pbio-1000031-b041] Debiais-Thibaud M, Borday-Birraux V, Germon I, Bourrat F, Metcalfe CJ (2007). Development of oral and pharyngeal teeth in the medaka (Oryzias latipes): comparison of morphology and expression of eve1 gene. J Exp Zool B: Mol Dev Evol.

[pbio-1000031-b042] Borday-Birraux V, Van der Heyden C, Debiais-Thibaud M, Verreijdt L, Stock DW (2006). Expression of Dlx genes during the development of the zebrafish pharyngeal dentition: evolutionary implications. Evol Dev.

[pbio-1000031-b043] Jackman WR, Draper BW, Stock DW (2004). Fgf signaling is required for zebrafish tooth development. Dev Biol.

[pbio-1000031-b044] Wise SB, Stock DW (2006). Conservation and divergence of Bmp2a, Bmp2b, and Bmp4 expression patterns within and between dentitions of teleost fishes. Evol Dev.

[pbio-1000031-b045] Schilling TF, Kimmel CB (1997). Musculoskeletal patterning in the pharyngeal segments of the zebrafish embryo. Development.

[pbio-1000031-b046] Harris MP, Rohner N, Schwarz H, Perathoner S, Konstantinidis P (2008). Zebrafish eda and edar mutants reveal conserved and ancestral roles of ectodysplasin signaling in vertebrates. PLoS Genet.

[pbio-1000031-b047] Fryer G, Illes TD (1972). The cichlid fishes of the Great Lakes of Africa: their biology and evolution.

[pbio-1000031-b048] Liem KF, Greenwood PH (1981). A functional approach to the phylogeny of the pharyngognath teleosts. Amer Zool.

[pbio-1000031-b049] Fraser GJ, Bloomquist RF, Streelman JT (2008). A periodic pattern generator for dental diversity. BMC Biology.

[pbio-1000031-b050] Loh YHE, Katz LS, Mims MC, Kocher TD, Yi S (2008). Comparative analysis reveals signatures of differentiation amid genomic polymorphism in Lake Malawi cichlids. Genome Biol.

[pbio-1000031-b051] Mikkola ML, Pispa J, Pekkanen M, Paulin L, Nieminen P (1999). Ectodysplasin, a protein required for epithelial morphogenesis, is a novel TNF homologue and promotes cell-matrix adhesion. Mech Dev.

[pbio-1000031-b052] Tucker AS, Headon DJ, Courtney JM, Overbeek P, Sharpe PT (2004). The activation level of the TNF family receptor, Edar, determines cusp number and tooth number during tooth development. Dev Biol.

[pbio-1000031-b053] Tucker AS, Headon DJ, Schneider P, Ferguson BM, Overbeek P (2000). Edar/Eda interactions regulate enamel knot formation in tooth morphogenesis. Development.

[pbio-1000031-b054] Mustonen T, Ilmonen M, Pummila M, Kangas AT, Laurikkala J (2004). Ectodysplasin A1 promotes placodal cell fate during early morphogenesis of ectodermal appendages. Development.

[pbio-1000031-b055] Laurikkala J, Mikkola M, Mustonen T, Aberg T, Koppinen P (2001). TNF signaling via the ligand-receptor pair ectodysplasin and edar controls the function of epithelial signaling centers and is regulated by Wnt and activin during tooth organogenesis. Dev Biol.

[pbio-1000031-b056] Laurikkala J, Pispa J, Jung HS, Nieminen P, Mikkola M (2002). Regulation of hair follicle development by the TNF signal ectodysplasin and its receptor Edar. Development.

[pbio-1000031-b057] Hashimoto I, Goto M, Kodera H, Inoue K (1976). The distribution of the teeth in the pharynx of salmonoid fishes. J Stomatol Soc Japan.

[pbio-1000031-b058] Hashimoto I, Goto M, Kodera H, Inoue K (1976). Two groups of teeth in the pharynx of the rainbow trout (Salmo gairdneri irideus GIBBONS). Jap J Oral Biol.

[pbio-1000031-b059] Smith MM, Coates MI (1998). Evolutionary origins of the vertebrate dentition: phylogenetic patterns and developmental evolution. Eur J Oral Sci.

[pbio-1000031-b060] Smith MM, Fraser GJ, Mitsiadis T (2009). Dental lamina as source of odontogenic stem cells: evolutionary origins and developmental control of tooth generation in gnathostomes. J Exp Zool B Mol Dev Evol.

[pbio-1000031-b061] Prince V (2002). The Hox Paradox: more complex(es) than imagined. Dev Biol.

[pbio-1000031-b062] Salsi V, Vigano MA, Cocchiarella F, Mantovani R, Zappavigna V (2008). Hoxd13 binds in vivo and regulates the expression of genes acting in key pathways for early limb and skeletal patterning. Dev Biol.

[pbio-1000031-b063] Tucker AS, Matthews KL, Sharpe PT (1998). Transformation of tooth type induced by inhibition of BMP signaling. Science.

[pbio-1000031-b064] Trumpp A, Depew MJ, Rubenstein JL, Bishop JM, Martin GR (1999). Cre-mediated gene inactivation demonstrates that FGF8 is required for cell survival and patterning of the first branchial arch. Genes Dev.

[pbio-1000031-b065] Peters H, Neubuser A, Balling R (1998). Pax genes and organogenesis: Pax9 meets tooth development. Eur J Oral Sci.

[pbio-1000031-b066] Peters H, Neubuser A, Kratochwil K, Balling R (1998). Pax9-deficient mice lack pharyngeal pouch derivatives and teeth and exhibit craniofacial and limb abnormalities. Genes Dev.

[pbio-1000031-b067] Donoghue PC, Sansom IJ, Downs JP (2006). Early evolution of vertebrate skeletal tissues and cellular interactions, and the canalization of skeletal development. J Exp Zool B: Mol Dev Evol.

[pbio-1000031-b068] Donoghue PC, Sansom IJ (2002). Origin and early evolution of vertebrate skeletonization. Microsc Res Tech.

[pbio-1000031-b069] Laurenti P, Thaeron C, Allizard F, Huysseune A, Sire JY (2004). Cellular expression of eve1 suggests its requirement for the differentiation of the ameloblasts and for the initiation and morphogenesis of the first tooth in the zebrafish (Danio rerio). Dev Dyn.

[pbio-1000031-b070] Reif W-E (1982). Evolution of dermal skeleton and dentition in vertebrates: the odontode-regulation theory. Evol Biol.

[pbio-1000031-b071] Kuratani S (2004). Evolution of the vertebrate jaw: comparative embryology and molecular developmental biology reveal the factors behind evolutionary novelty. J Anat.

[pbio-1000031-b072] Chen Y, Zhang Y, Jiang TX, Barlow AJ, St Amand TR (2000). Conservation of early odontogenic signaling pathways in Aves. Proc Natl Acad Sci U S A.

[pbio-1000031-b073] Johanson Z, Smith MM (2005). Origin and evolution of gnathostome dentitions: a question of teeth and pharyngeal denticles in placoderms. Biol Rev.

[pbio-1000031-b074] Smith MM, Johanson Z (2003). Separate evolutionary origins of teeth from evidence in fossil jawed vertebrates. Science.

[pbio-1000031-b075] Johanson Z, Smith MM (2003). Placoderm fishes, pharyngeal denticles, and the vertebrate dentition. J Morphol.

[pbio-1000031-b076] Lumsden AG (1988). Spatial organization of the epithelium and the role of neural crest cells in the initiation of the mammalian tooth germ. Development.

[pbio-1000031-b077] Tummers M, Thesleff I (2003). Root or crown: a developmental choice orchestrated by the differential regulation of the epithelial stem cell niche in the tooth of two rodent species. Development.

[pbio-1000031-b078] Wang XP, Suomalainen M, Felszeghy S, Zelarayan LC, Alonso MT (2007). An integrated gene regulatory network controls stem cell proliferation in teeth. PLoS Biol.

[pbio-1000031-b079] Jarvinen E, Salazar-Ciudad I, Birchmeier W, Taketo MM, Jernvall J (2006). Continuous tooth generation in mouse is induced by activated epithelial Wnt/beta-catenin signaling. Proc Natl Acad Sci U S A.

[pbio-1000031-b080] Harris MP, Fallon JF, Prum RO (2002). Shh-Bmp2 signaling module and the evolutionary origin and diversification of feathers. J Exp Zool.

[pbio-1000031-b081] Houghton L, Lindon C, Morgan BA (2005). The ectodysplasin pathway in feather tract development. Development.

[pbio-1000031-b082] Lin CM, Jiang TX, Widelitz RB, Chuong CM (2006). Molecular signaling in feather morphogenesis. Curr Opin Cell Biol.

[pbio-1000031-b083] Pispa J, Thesleff I (2003). Mechanisms of ectodermal organogenesis. Dev Biol.

[pbio-1000031-b084] Pummila M, Fliniaux I, Jaatinen R, James MJ, Laurikkala J (2007). Ectodysplasin has a dual role in ectodermal organogenesis: inhibition of Bmp activity and induction of Shh expression. Development.

[pbio-1000031-b085] Sire JY, Akimenko MA (2004). Scale development in fish: a review, with description of sonic hedgehog (shh) expression in the zebrafish (Danio rerio). Int J Dev Biol.

[pbio-1000031-b086] Thesleff I, Vaahtokari A, Partanen A-M (1995). Regulation of organogenesis. Common molecular mechanisms regulating the development of teeth and other organs. Int J Dev Biol.

[pbio-1000031-b087] Casal ML, Jezyk PF, Greek JM, Goldschmidt MH, Patterson DF (1997). X-linked ectodermal dysplasia in the dog. J Hered.

[pbio-1000031-b088] Clarke A (1987). Hypohidrotic ectodermal dysplasia. J Med Genet.

[pbio-1000031-b089] Drogemuller C, Distl O, Leeb T (2001). Partial deletion of the bovine ED1 gene causes anhidrotic ectodermal dysplasia in cattle. Genome Res.

[pbio-1000031-b090] Plikus MV, Zeichner-David M, Mayer JA, Reyna J, Bringas P (2005). Morphoregulation of teeth: modulating the number, size, shape and differentiation by tuning Bmp activity. Evol Dev.

[pbio-1000031-b091] Streelman JT, Peichel CL, Parichy DM (2007). Developmental genetics of adaptation in fishes: the case for novelty. Annu Rev Ecol Evol Syst.

[pbio-1000031-b092] Cohn MJ, Lovejoy CO, Wolpert L, Coates MI (2002). Branching, segmentation and the metapterygial axis: pattern versus process in the vertebrate limb. Bioessays.

[pbio-1000031-b093] Cohn MJ, Tickle C (1999). Developmental basis of limblessness and axial patterning in snakes. Nature.

[pbio-1000031-b094] Freitas R, Zhang G, Cohn MJ (2007). Biphasic Hoxd gene expression in shark paired fins reveals an ancient origin of the distal limb domain. PLoS ONE.

[pbio-1000031-b095] Litingtung Y, Dahn RD, Li Y, Fallon JF, Chiang C (2002). Shh and Gli3 are dispensable for limb skeleton formation but regulate digit number and identity. Nature.

[pbio-1000031-b096] Matsuoka T, Ahlberg PE, Kessaris N, Iannarelli P, Dennehy U (2005). Neural crest origins of the neck and shoulder. Nature.

[pbio-1000031-b097] Shubin N, Tabin C, Carroll S (1997). Fossils, genes and the evolution of animal limbs. Nature.

[pbio-1000031-b098] Sordino P, van der Hoeven F, Duboule D (1995). Hox gene expression in teleost fins and the origin of vertebrate digits. Nature.

[pbio-1000031-b099] Posada D, Crandall KA (1998). MODELTEST: testing the model of DNA substitution. Bioinformatics.

[pbio-1000031-b100] Ronquist F, Huelsenbeck JP (2003). Mr. Bayes 3: Bayesian phylogenetic inference under mixed models. Bioinformatics.

[pbio-1000031-b101] Hulsey CD, Mims MC, Streelman JT (2007). Do constructional constraints influence cichlid craniofacial diversification. Proc Biol Sci.

[pbio-1000031-b102] Felsenstein J (1985). Phylogenies and the comparative method. Am Nat.

[pbio-1000031-b103] Kocher TD, Conroy JA, Mckaye KR, Stauffer JR, Lockwood SF (1995). Evolution of NADH dehydrogenase subunit 2 in East African cichlid fish. Mol Phylog Evol.

[pbio-1000031-b104] Streelman JT, Albertson RC, Kocher TD (2007). Variation in body size and trophic morphology within and among genetically differentiated populations of the cichlid fish, Metriaclima zebra, from Lake Malawi. Freshw Biol.

[pbio-1000031-b105] Hoegg S, Boore JL, Kuehl JV, Meyer A (2007). Comparative phylogenomic analyses of teleost fish Hox gene clusters: lessons from the cichlid fish Astatotilapia burtoni. BMC Genomics.

[pbio-1000031-b106] Thesleff I, Sharpe P (1997). Signalling networks regulating dental development. Mech Dev.

[pbio-1000031-b107] Tucker AS, Al Khamis A, Sharpe PT (1998). Interactions between Bmp-4 and Msx-1 act to restrict gene expression to odontogenic mesenchyme. Dev Dyn.

[pbio-1000031-b108] Thomas BL, Liu JK, Rubenstein JL, Sharpe PT (2000). Independent regulation of Dlx2 expression in the epithelium and mesenchyme of the first branchial arch. Development.

[pbio-1000031-b109] Thomas BL, Porteus MH, Rubenstein JL, Sharpe PT (1995). The spatial localization of Dlx-2 during tooth development. Connect Tissue Res.

[pbio-1000031-b110] Mitsiadis TA, Mucchielli ML, Raffo S, Proust JP, Koopman P (1998). Expression of the transcription factors Otlx2, Barx1 and Sox9 during mouse odontogenesis. Eur J Oral Sci.

[pbio-1000031-b111] Mucchielli ML, Mitsiadis TA, Raffo S, Brunet JF, Proust JP (1997). Mouse Otlx2/RIEG expression in the odontogenic epithelium precedes tooth initiation and requires mesenchyme-derived signals for its maintenance. Dev Biol.

[pbio-1000031-b112] St Amand TR, Zhang Y, Semina EV, Zhao X, Hu Y (2000). Antagonistic signals between BMP4 and FGF8 define the expression of Pitx1 and Pitx2 in mouse tooth-forming anlage. Dev Biol.

[pbio-1000031-b113] Aberg T, Wang XP, Kim JH, Yamashiro T, Bei M (2004). Runx2 mediates FGF signaling from epithelium to mesenchyme during tooth morphogenesis. Dev Biol.

[pbio-1000031-b114] Cobourne MT, Hardcastle Z, Sharpe PT (2001). Sonic hedgehog regulates epithelial proliferation and cell survival in the developing tooth germ. J Dent Res.

[pbio-1000031-b115] Cobourne MT, Miletich I, Sharpe PT (2004). Restriction of sonic hedgehog signalling during early tooth development. Development.

[pbio-1000031-b116] Sarkar L, Cobourne M, Naylor S, Smalley M, Dale T (2000). Wnt/Shh interactions regulate ectodermal boundary formation during mammalian tooth development. Proc Natl Acad Sci U S A.

[pbio-1000031-b117] Sarkar L, Sharpe PT (1999). Expression of Wnt signalling pathway genes during tooth development. Mech Dev.

[pbio-1000031-b118] Streelman JT, Albertson RC (2006). Evolution of novelty in the cichlid dentition. J Exp Zool B: Mol Dev Evol.

