# Supplementary material for: An Ancient Gene Network Is Co-opted for Teeth on Old and New Jaws
Source: PLoS Biol. 2009 Feb 10;7(2):e1000031. doi: 10.1371/journal.pbio.1000031 (PMC2637924; doi:10.1371/journal.pbio.1000031)
Supplement: Supplementary file 1 [file pbio.1000031.st001.doc]

Supplementary Information

| **Malawi Cichlids species** | **Pharyngeal** | **Oral** | **SL** |
| --- | --- | --- | --- |
| *Astatotilapia calliptera* | 178 | 176 | 73 |
| *Buccochromis heterotaenia* | 163 | 228 | 116 |
| *Chilotilapia euchilus* | 227 | 128 | 89 |
| *Copadichromis eucinostomus* | 300 | 132 | 85 |
| *Copadichromis mbenji* | 235 | 137 | 87 |
| *Copadichromis quadrimaculatus* | 410 | 129 | 92 |
| *Cynotilapia afra* | 314 | 67 | 75 |
| *Cyphotilapia moorii* | 154 | 223 | 131 |
| *Dimidiochromis compressiceps* * | 113 | 66 | 124 |
| *Dimidiochromis kiwinge* | 190 | 177 | 191 |
| *Docimodus evelynae* | 106 | 90 | 59 |
| *Fossorochromis rostratus* | 164 | 138 | 90 |
| *Labeotropheus fuelleborni* * | 602 | 497 | 98 |
| *Labeotropheus trewavasae* | 429 | 376 | 87 |
| *Labidochromis gigas* | 244 | 125 | 72 |
| *Melanochromis auratus* | 312 | 375 | 72 |
| *Melanochromis vermivorous* | 308 | 230 | 73 |
| *Metriaclima aurora* | 602 | 210 | 68 |
| *Metriaclima livingstoni* | 372 | 152 | 138 |
| *Metriaclima zebra* * | 448 | 176 | 76 |
| *Nimbochromis linni* | 126 | 173 | 79 |
| *Nimbochromis polystigma* | 150 | 144 | 101 |
| *Otopharynx pictus "maleri"* | 229 | 141 | 91 |
| *Otopharynx walteri* | 198 | 163 | 89 |
| *Petrotilapia nigra* | 722 | 1170 | 96 |
| *Placidochromis milomo* | 226 | 179 | 97 |
| *Protomelas ornatus* | 169 | 208 | 149 |
| *Protomelas spilopterus "blue"* | 140 | 88 | 85 |
| *Rhamphochromis esox* | 110 | 65 | 63 |
| *Stigmatochromis woodi* | 334 | 194 | 120 |
| *Taeniolethrinops praeorbitalis* | 271 | 300 | 91 |
| *Trematocranus placodon* | 88 | 196 | 141 |
| *Tropheops "orange chest"* | 314 | 253 | 80 |
| *Tropheops gracilior* | 478 | 349 | 72 |
| *Tropheops microstoma* | 296 | 293 | 170 |
| *Tyrannochromis macrostoma* | 120 | 178 | 92 |
| *Tyrannochromis nigriventer* | 116 | 264 | 95 |

**Supplementary Table 1**: Mean oral and pharyngeal tooth counts and standard length (SL) for one – four Malawi cichlid adults from 37 species. *D. compressiceps*, *M. zebra*, and *L. fuelleborni* (asterisks) are indicated on Figure 2A-K.
