# Supplementary material for: An Ancient Gene Network Is Co-opted for Teeth on Old and New Jaws
Source: PLoS Biol. 2009 Feb 10;7(2):e1000031. doi: 10.1371/journal.pbio.1000031 (PMC2637924; doi:10.1371/journal.pbio.1000031)
Supplement: Supplementary file 2 [file pbio.1000031.st002.doc]

| Genetic Interaction from Figure 7(4) | Citation |
| --- | --- |
| 1. bmp4🡪shh | [1] |
| 2. bmp4🡪dlx2 | [2] |
| 3. bmp4-/barx1 | [3] |
| 4. bmp4-/pax9 | [4, 5] |
| 5. bmp2-/pax9 | [5, 6] |
| 6. pitx2/-/bmp4 | [7] |
| 7. eda🡪shh | [8] |
| 8. edar🡨🡪shh | Hair/Feather [9] |
| 9. eda🡪edar | Tooth and salivary gland [10, 11] |
| 10. hox🡪shh | Inferred [12] |
| 11. hox🡪barx1 | Inferred [13] |
| 12. hox🡪runx2 | Inferred [14, 15] |
| 13. runx2🡨🡪fgf | [16] |
| 14. fgf🡪pax9 | [4, 5] |
| 15. fgf🡪barx1 | [3] |
| 16. fgf🡪dlx2 | [2] |
| 17. fgf🡪pitx2 | [7] |
| 18. wnt/Bcat-/shh | [17] |
| 19. runx2🡪shh | [16] |

**Supplementary Table 2:** Referenced molecular interactions during tooth development, as shown in Figure 7(4).

1.	Y Zhang, Z Zhang, X Zhao, X Yu, Y Hu, B Geronimo, SH Fromm, YP Chen: **A new function of BMP4: dual role for BMP4 in regulation of Sonic hedgehog expression in the mouse tooth germ**. *Development* 2000, **127**:1431-43.

2.	BL Thomas, JK Liu, JL Rubenstein, PT Sharpe: **Independent regulation of Dlx2 expression in the epithelium and mesenchyme of the first branchial arch**. *Development* 2000, **127**:217-24.

3.	AS Tucker, KL Matthews, PT Sharpe: **Transformation of tooth type induced by inhibition of BMP signaling**. *Science* 1998, **282**:1136-8.

4.	H Peters, A Neubuser, K Kratochwil, R Balling: **Pax9-deficient mice lack pharyngeal pouch derivatives and teeth and exhibit craniofacial and limb abnormalities**. *Genes Dev* 1998, **12**:2735-47.

5.	A Neubuser, H Peters, R Balling, GR Martin: **Antagonistic interactions between FGF and BMP signaling pathways: a mechanism for positioning the sites of tooth formation**. *Cell* 1997, **90**:247-55.

6.	S Vainio, I Karavanova, A Jowett, I Thesleff: **Identification of BMP-4 as a signal mediating secondary induction between epithelial and mesenchymal tissues during early tooth development**. *Cell* 1993, **75**:45-58.

7.	TR St Amand, Y Zhang, EV Semina, X Zhao, Y Hu, L Nguyen, JC Murray, Y Chen: **Antagonistic signals between BMP4 and FGF8 define the expression of Pitx1 and Pitx2 in mouse tooth-forming anlage**. *Dev Biol* 2000, **217**:323-32.

8.	M Pummila, I Fliniaux, R Jaatinen, MJ James, J Laurikkala, P Schneider, I Thesleff, ML Mikkola: **Ectodysplasin has a dual role in ectodermal organogenesis: inhibition of Bmp activity and induction of Shh expression**. *Development* 2007, **134**:117-25.

9.	R Schmidt-Ullrich, DJ Tobin, D Lenhard, P Schneider, R Paus, C Scheidereit: **NF-kappaB transmits Eda A1/EdaR signalling to activate Shh and cyclin D1 expression, and controls post-initiation hair placode down growth**. *Development* 2006, **133**:1045-57.

10.	AS Tucker, DJ Headon, P Schneider, BM Ferguson, P Overbeek, J Tschopp, PT Sharpe: **Edar/Eda interactions regulate enamel knot formation in tooth morphogenesis**. *Development* 2000, **127**:4691-700.

11.	J Pispa, ML Mikkola, T Mustonen, I Thesleff: **Ectodysplasin, Edar and TNFRSF19 are expressed in complementary and overlapping patterns during mouse embryogenesis**. *Gene Expr Patterns* 2003, **3**:675-9.

12.	V Knezevic, R De Santo, K Schughart, U Huffstadt, C Chiang, KA Mahon, S Mackem: **Hoxd-12 differentially affects preaxial and postaxial chondrogenic branches in the limb and regulates Sonic hedgehog in a positive feedback loop**. *Development* 1997, **124**:4523-36.

13.	V Salsi, MA Vigano, F Cocchiarella, R Mantovani, V Zappavigna: **Hoxd13 binds in vivo and regulates the expression of genes acting in key pathways for early limb and skeletal patterning**. *Dev Biol* 2008, **317**:497-507.

14.	MQ Hassan, S Saini, JA Gordon, AJ van Wijnen, M Montecino, JL Stein, GS Stein, JB Lian: **Molecular Switches Involving Homeodomain Proteins, HOXA10 and RUNX2 Regulate Osteoblastogenesis**. *Cells Tissues Organs* 2008.

15.	MQ Hassan, R Tare, SH Lee, M Mandeville, B Weiner, M Montecino, AJ van Wijnen, JL Stein, GS Stein, JB Lian: **HOXA10 controls osteoblastogenesis by directly activating bone regulatory and phenotypic genes**. *Mol Cell Biol* 2007, **27**:3337-52.

16.	T Aberg, XP Wang, JH Kim, T Yamashiro, M Bei, R Rice, HM Ryoo, I Thesleff: **Runx2 mediates FGF signaling from epithelium to mesenchyme during tooth morphogenesis**. *Dev Biol* 2004, **270**:76-93.

17.	L Sarkar, M Cobourne, S Naylor, M Smalley, T Dale, PT Sharpe: **Wnt/Shh interactions regulate ectodermal boundary formation during mammalian tooth development**. *Proc Natl Acad Sci U S A* 2000, **97**:4520-4.
